# Hominy

**Published:** 1855-03

**Authors:** 


Hominy.—We know the value of the article as an economical, palatable, wholesome, nutricious food; and we wish we could induce every one of our readers to try it, as we do every morning for breakfast. Hominy is coming more and more into use in this city every year, but not half so much as it would if better known, and particularly if our cooks knew how to prepare it. Nothing can be more simple, and that perhaps is the reason, because it is so simple nobody can understand it. We give the formula:—

Wash the hominy if you think you must—though we should as soon think of washing flour before using it—and put it in soak in three times as much water as you wish to cook of hominy, and set it where it will become a little warm. It should soak at least twelve hours. Boil it in the same water in a porcelain lined kettle, until it is soft, still leaving each grain quite whole. Be very careful to keep sufficient water in the kettle to prevent the mass from sticking, or it will burn. When done, all the water will be absorbed. Never add salt, or butter, or meat to the hominy while soaking. Season it after it is done, or leave every one to add salt, sugar, butter, or meat gravy to his liking.

In this city, the article thus made is called samp, though verry erroneously; and the name of hominy only given to the product of a grinding mill, which cracks the corn, which is afterwards winnowed of the hulls, and sifted into different degrees of coarseness. The coarsest is always best. It costs at present about three cents a pound; it is cheaper and better than rice; it is a good substitute for potatoes; and $3 worth of hominy will go further than $10 worth of potatoes.—*Tribune.*

